# Regulation of Respiration and Apoptosis by Cytochrome *c* Threonine 58 Phosphorylation

**DOI:** 10.1038/s41598-019-52101-z

**Published:** 2019-11-01

**Authors:** Junmei Wan, Hasini A. Kalpage, Asmita Vaishnav, Jenney Liu, Icksoo Lee, Gargi Mahapatra, Alice A. Turner, Matthew P. Zurek, Qinqin Ji, Carlos T. Moraes, Maurice-Andre Recanati, Lawrence I. Grossman, Arthur R. Salomon, Brian F. P. Edwards, Maik Hüttemann

**Affiliations:** 1 Center for Molecular Medicine and Genetics, Wayne State University, Detroit, MI 48201 USA; 2 Department of Biochemistry, Microbiology and Immunology, Wayne State University, Detroit, MI 48201 USA; 3 College of Medicine, Dankook University, Cheonan-si, Chungcheongnam-do 31116 Republic of Korea; 4 MCB Department, Brown University, Providence, RI 02912 USA; 5 Department of Neurology, University of Miami School of Medicine, Miami, FL 33136 USA; 6 Department of Obstetrics and Gynecology, Wayne State University, Detroit, MI 48201 USA; 7 Present Address: Department of Internal Medicine, Section on Gerontology and Geriatric Medicine, Wake Forest University Health Sciences, Winston-Salem, NC 27157 USA

**Keywords:** Mitochondrial proteins, Phosphorylation

## Abstract

Cytochrome *c* (Cyt*c*) is a multifunctional protein, acting as an electron carrier in the electron transport chain (ETC), where it shuttles electrons from *bc*_1_ complex to cytochrome *c* oxidase (COX), and as a trigger of type II apoptosis when released from the mitochondria. We previously showed that Cyt*c* is regulated in a highly tissue-specific manner: Cyt*c* isolated from heart, liver, and kidney is phosphorylated on Y97, Y48, and T28, respectively. Here, we have analyzed the effect of a new Cyt*c* phosphorylation site, threonine 58, which we mapped in rat kidney Cyt*c* by mass spectrometry. We generated and overexpressed wild-type, phosphomimetic T58E, and two controls, T58A and T58I Cyt*c*; the latter replacement is found in human and testis-specific Cyt*c*. *In vitro*, COX activity, caspase-3 activity, and heme degradation in the presence of H_2_O_2_ were decreased with phosphomimetic Cyt*c* compared to wild-type. Cyt*c*-knockout cells expressing T58E or T58I Cyt*c* showed a reduction in intact cell respiration, mitochondrial membrane potential (∆Ψ_m_), ROS production, and apoptotic activity compared to wild-type. We propose that, under physiological conditions, Cyt*c* is phosphorylated, which controls mitochondrial respiration and apoptosis. Under conditions of stress Cyt*c* phosphorylations are lost leading to maximal respiration rates, ∆Ψ_m_ hyperpolarization, ROS production, and apoptosis.

## Introduction

Cytochrome *c* (Cyt*c*) is a small, globular nuclear-encoded protein with a covalently attached heme group. It is located at the mitochondrial intermembrane space (IMS) as a mobile single electron carrier between Complexes III (*bc*_1_ complex) and IV (cytochrome *c* oxidase, COX) of the electron transport chain (ETC). Cyt*c* null mice die at midgestation^1^ when energy metabolism switches from mainly glycolytic to mainly aerobic^2^, indicating that Cyt*c* is an essential protein for the development of organisms and indispensable for mitochondrial ATP production. In addition to functioning in mitochondrial respiration, under stress conditions Cyt*c* serves as a proapoptotic signal^3^. Upon its release from the mitochondria into the cytosol, Cyt*c* interacts with apoptotic protease-activating factor 1 (Apaf-1) to form the apoptosome, leading to activation of caspase-9 and the downstream caspase cascade^3,4^. Beyond these two major functions, Cyt*c* acts as a peroxidase that oxidizes the mitochondrial membrane lipid cardiolipin at earlier stages of apoptosis, thus promoting Cyt*c* release from the IMS into the cytosol^5^. In addition, Cyt*c* can function as both a reactive oxygen species (ROS) scavenger and producer via reduction of p66^shc^, a protein involved in ROS generation and apoptosis^6^. Furthermore, Cyt*c* serves as the electron acceptor in the Erv1-Mia40 redox relay system involved in mitochondrial IMS protein import^7^.

Considering the multiple functions that Cyt*c* plays in different pathophysiological conditions, it is imperative that Cyt*c* is tightly regulated. Earlier, the study of regulatory mechanisms of Cyt*c* mainly focused on allosteric regulation by binding of ATP and the expressions of the tissue-specific (somatic and testis) isoforms in mammals^8,9^. Recently, a novel regulatory mechanism has emerged, and we reported that Cyt*c* can be phosphorylated in a tissue-specific manner, which appears to be physiologically relevant because the majority of the Cyt*c* pool was found to be modified when the protein was purified under conditions preserving the physiological phosphorylation status (reviewed in^10^). Under basal physiological conditions, Cyt*c* was phosphorylated on Y97 in heart, Y48 in liver, and T28 in kidney^11–13^. Phosphorylation of T28 and S47 was also found in skeletal muscle^14,15^. These phosphorylations can be lost under stressed conditions, such as ischemia. Functional studies showed that phosphorylation or phosphomimetic substitution of Cyt*c* T28, Y48, and Y97 leads to a partial inhibition of respiration and reduced mitochondrial membrane potential (ΔΨ_m_) levels and ROS production^11–13^. We then proposed that this mechanism maintains optimal intermediate ΔΨ_m_ levels for efficient ATP generation but limits ROS production, which occurs at pathologically high ΔΨ_m_ levels^10^.

Interestingly, those phosphorylated or phosphomimetic Cyt*c* species have different effects on caspase activation. For example, Y48E abolishes the capacity of triggering apoptosis and fully protects against caspase activation whereas T28E has no such effect^13,16^. Therefore, phosphorylations at different sites exert distinct effects on the regulation of Cyt*c* functions to meet tissue-specific energy needs and regulation of apoptosis.

Based on our previous studies we have proposed a model in which the activity of the ETC is controlled by phosphorylations of Cyt*c* under normal conditions, thus preventing ΔΨ_m_ hyperpolarization and subsequent excessive ROS production that causes apoptosis. We here characterize a new phosphorylation of Cyt*c* on T58 that we found in rat kidney tissue under normal conditions, suggesting that it plays a biological role. Additionally, previous research concluded that this site is important for the binding of cardiolipin to Cyt*c*^17^. Therefore, we hypothesized that T58 phosphorylation may cause structural and functional changes. To further test our hypothesis and model, we analyzed the effects of this new Cyt*c* phosphorylation site. *In vitro*, phosphomimetic T58E substitution causes an inhibition of respiration in the reaction with COX and reduced downstream caspase-3 activity. In a cell culture model, introduction of phosphomimetic T58E and WT Cyt*c* into Cyt*c* knockout cells revealed that phosphomimetic substitution leads to reduced intact cell respiration, ΔΨ_m_, and ROS production. Moreover, this substitution protects the cells from apoptosis when challenged with hydrogen peroxide (H_2_O_2_) or staurosporine. These data suggest that Cyt*c* phosphorylation on T58 regulates the overall flux of the ETC, preventing ΔΨ_m_ hyperpolarization and subsequent excessive ROS production that causes apoptosis^10^.

## Results

### Rat kidney cytochrome *c* is phosphorylated on threonine 58

**Figure 1:**
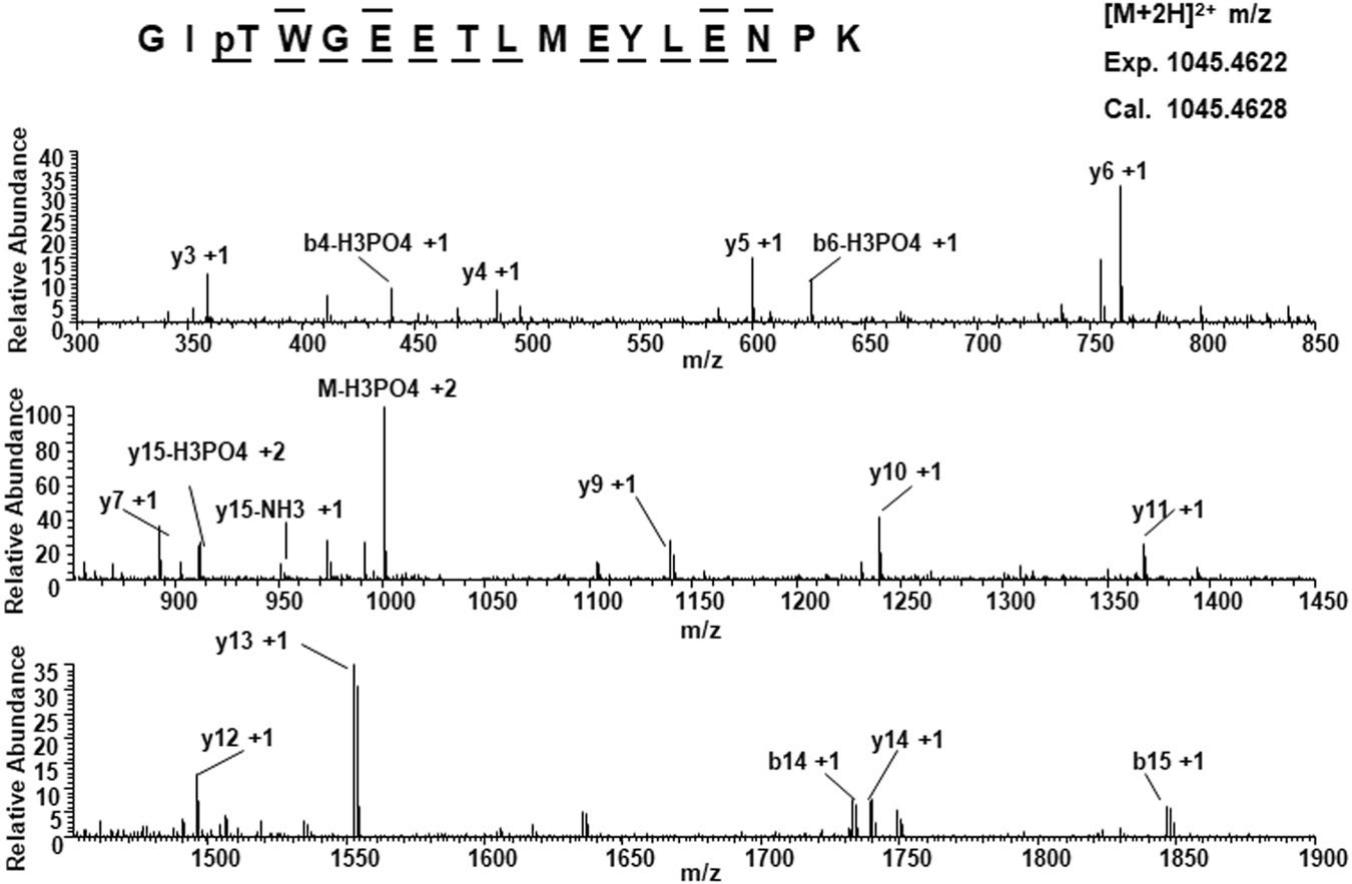
Kidney Cyt*c* is phosphorylated on T58. Nano-LC/ES/MS/MS spectrum of GIpTWEETLMEYLENPK shows phosphorylation on T58. The phosphorylation site was unambiguously assigned by fragment ions y14 and y15. The sequence of the peptide was assigned by b6, b8, b14, b15, y3, y4, y5, y6, y7, y9, y10, y11, y12, y13, y14, y15.

We purified Cyt*c* from kidney tissue under conditions preserving physiological phosphorylations. Previously, we reported T28 phosphorylation of Cyt*c* purified from kidney^13^, an organ that heavily relies on aerobic energy metabolism. Surprisingly, mass spectrometric analysis revealed a new phosphorylation site, on T58, in two out of five independent kidney preparations (Fig. 1), while T28 was identified in all of them. The T58 residue is conserved in the somatic Cyt*c* isoform of mammals but is replaced with an isoleucine in the testis isoform.

### Overexpression and purification of functional cytochrome *c* variants in *E. coli* cells

**Figure 2:**
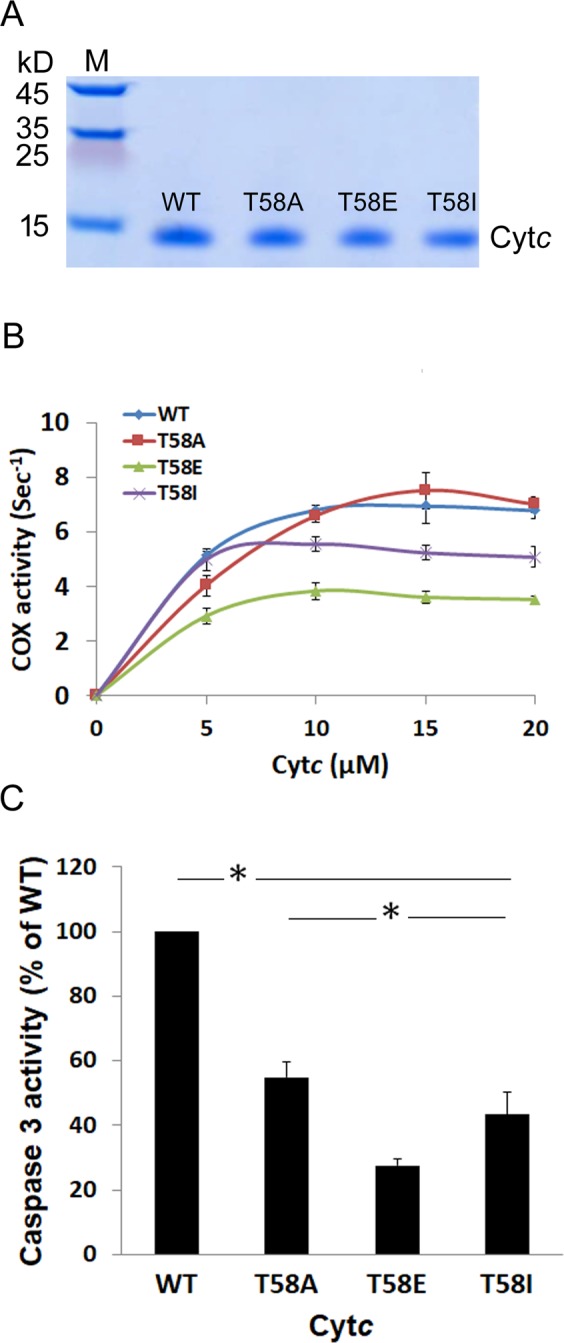
COX and Caspase-3 activity are reduced in the reaction with purified recombinant phosphomimetic Cyt*c*. (**A**) Coomassie blue-stained gel confirming the purity of recombinant Cyt*c* overexpressed and purified from bacteria. All four Cyt*c* variants were purified and displayed a single band on a 10% Tris-Tricine SDS-PAGE gel. (**B**) Oxygen consumption of cow liver COX (150 nM) was measured using the Oxygraph system (Hansatech) at concentrations of 0, 5, 10, 15, and 20 µM of purified WT, T58A, T58E, and T58I Cyt*c*. Data are expressed as turnover number (s^-1^). (**C**) Cytosolic extracts of Cyt*c* knockout embryonic fibroblast cells at a total protein concentration of 1 mg/mL were incubated with Cyt*c* variants at a concentration of 20 µg/mL in a total volume of 10 µL at 37 °C for 2 h. Rhodamine fluorescence was analyzed after caspase-3-mediated cleavage of the artificial substrate DEVD-R110. Data are presented as percentage of WT Cyt*c* and expressed as means ± SEM. T58E Cyt*c* shows 70% reduced caspase-3 activity. *p < 0.05.

To study the effect of T58 phosphorylation *in vitro* we generated T58E phosphomimetic Cyt*c*. Phosphomimetic amino acid replacement can functionally mimic protein phosphorylation and be used to model the functional effects of fully phosphorylated proteins^18,19^. We and others have applied this approach to Cyt*c* by replacing phosphorylatable Cyt*c* residues with the negatively charged amino acid glutamate, which produced similar functional effects as *in vivo*-phosphorylated Cyt*c*^13,16,20^. We therefore constructed mouse Cyt*c* expression plasmids for WT and three mutants, including phosphomimetic T58E, and T58A as a non-phosphorylatable control. We also generated T58I Cyt*c* as an additional control because the isoleucine residue is found at this position in the testis isoform of mammals that express two isoforms of Cyt*c*. Furthermore, isoleucine is present in Cyt*c* of humans that lack tissue-specific isoforms and only express a single Cyt*c*. To obtain sufficient amounts of the proteins, we overexpressed these variants in *E. coli* C41 (DE3) cells. The Coomassie blue stained gel shows that Cyt*c* variants were purified to homogeneity (Fig. 2A) with a spectral ratio of the 410 nm/280 nm peaks greater than 4^16^.

### Cytochrome *c* oxidase activity is decreased in the reaction with phosphomimetic cytochrome *c*

Our previous studies showed that Y48E and T28E phosphomimetic Cyt*c* displayed an ~30% and 50% reduction, respectively, in the reaction with isolated bovine liver COX at maximal turnover similar to the reaction with Y48 and T28 phosphorylated Cyt*c*, suggesting that the replacement with glutamate is an ideal phosphomimetic model^13,16^. To understand if T58E, located at a different site, has a similar effect, we first performed polarography experiments to analyze the activities of the Cyt*c* variants in the reaction with purified cow liver COX. COX was isolated as a regulatory-competent enzyme under conditions preserving its *in vivo* phosphorylation status^21^. As shown in Fig. 2B, the oxygen consumption rates of T58E and T58I were reduced by 45% and 25% compared to the WT Cyt*c* whereas T58A showed no significant change. The apparent K_m_ values of the Cyt*c* variants in the reactions with COX were 3.0, 3.0, 4.5, and 2.5 µM for WT, T58E, T58A, and T58I Cyt*c*, respectively.

### Caspase-3 activity is reduced with phosphomimetic cytochrome *c*

To test the effect of T58E on apoptosis, we analyzed the ability of Cyt*c* variants to initiate apoptosis using a cell-free caspase-3 assay. The Cyt*c* variants were incubated with cytosolic extracts from Cyt*c* knockout embryonic fibroblasts^1^. We assessed downstream caspase-3 activation by measuring fluorescence intensity produced after cleavage of the artificial caspase-3 substrate DEVD conjugated with rhodamine. Interestingly, all the Cyt*c* mutants displayed reduced caspase-3 activity, by about 70%, 50%, and 60% for T58E, T58A, and T58I Cyt*c* compared to WT (Fig. 2C), suggesting lower levels of apoptosome formation in the presence of the mutants.

### Phosphomimetic cytochrome *c* displays similar thermal stability to WT cytochrome *c*

To determine the thermal stability of T58 variants of Cyt*c*, the recombinant proteins were gradually heated to undergo denaturation in the presence of SYPRO Orange dye, which interacts with hydrophobic regions of the protein that become exposed upon denaturation. The hydrophobic regions increase fluorescence emission of the dye resulting in a fluorescence readout for thermal denaturation of the protein^22^. The first melting temperature (T_m_) for WT, T58A, T58E, and T58I were 55.7 ± 0.3, 54.4 ± 1.1, 56.4 ± 0.9, and 91.5 ± 0.2 °C, respectively. WT, T58A, and T58E Cyt*c* showed second melting temperatures (T_m2_) of 91.2 ± 0.1, 91.5 ± 0.4, and 91.4 ± 0.5 °C, respectively (Supplementary Fig. 1). T58I displayed only one melting temperature whereas the other variants displayed two, suggesting that different domains of WT and T58E Cyt*c* have varying melting temperatures resulting in a step-wise unfolding. The results confirm that the T58I Cyt*c* replacement observed in the testis isoform of Cyt*c* and human Cyt*c* stabilizes the protein. These results are consistent with the heme degradation studies (see below), which also suggest that T58I is the most stable Cyt*c* variant.

### Phosphomimetic cytochrome *c* displays lower redox potential and lower oxidation and higher reduction rates and is partially resistant to heme degradation by hydrogen peroxide

**Figure 3:**
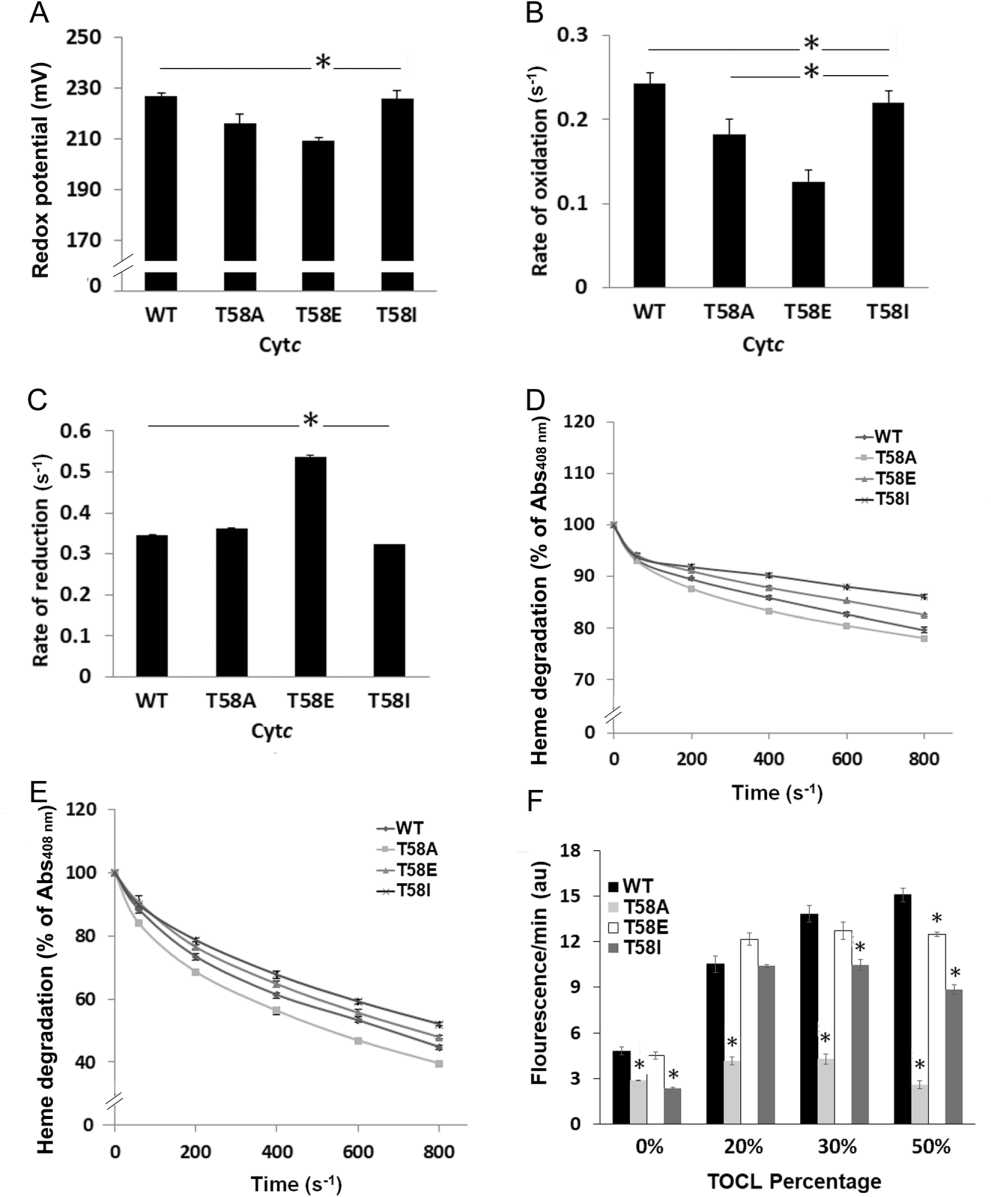
Phosphomimetic Cyt*c* displays distinct redox features, oxidative stability, and cardiolipin peroxidase activity. (**A**) Redox potential was measured in the presence of DCIP as reference compound. (**B**) Oxidation of ferro-Cyt*c* by H_2_O_2_ (100 µM) and (**C**), reduction of ferri-Cyt*c* by ascorbate (200 µM) were measured spectrophotometrically at 550 nm at 0, 10, 20, and 30 sec. (**D,E**) Heme degradation of ferri-Cyt*c* (**D**) and ferro-Cyt*c* (**E**) were measured as a decrease in absorption of the heme Soret band at 408 nm and recorded at 0, 60, 200, 400, 600, and 800 sec in the presence of excess H_2_O_2_ (3 mM). (**F**) Cardiolipin peroxidation. Liposomes containing 0%, 20%, 30%, and 50% of tetraoleoyl-cardiolipin (TOCL) and 1,2-Dioleoyl-sn-glycero-3-phosphocholine (DOPC) were used in the presence of Amplex Red to measure cardiolipin peroxidase activity of WT, T58A, T58E and T58I Cyt*c*(1 µM) in 20 mM K-HEPES buffer, pH 7.2. Fluorescence of resorufin (oxidation product of Amplex Red) was recorded using a Fluoroskan Ascent microplate reader with excitation/emission wavelengths of 530 nm/590 nm upon addition of Amplex Red (10 µM) and H_2_O_2_ (5 µM). Data are expressed as means ± SEM. *p < 0.05.

The redox midpoint potential of native Cyt*c* is between that of Complex III and Complex IV, allowing efficient electron transfer during respiration. The reported midpoint potential values for Cyt*c* are in the range of 220–270 mV. To determine the tendency of the Cyt*c* mutants to acquire electrons, redox potentials for WT, T58A, T58E, and T58I Cyt*c* were measured using the equilibration method. The redox potentials of the Cyt*c* variants were in a similar range from 209 mV (T58E) to 227 mV (WT) as shown in Fig. 3A.

Cyt*c* also functions as a ROS scavenger. Therefore, we were interested in understanding if the phosphomimetic substitution (T58E) exhibits a different behavior in reaction with an oxidant (H_2_O_2_) or a reductant (ascorbate). We analyzed oxidation rates of ferro-(Fe^2+^)-Cyt*c* variants in the presence of 100 µM H_2_O_2_ and reduction rates of ferri-(Fe^3+^)-Cyt*c* variants in the presence of 200 µM ascorbate. Interestingly, T58E Cyt*c* displayed a 50% lower oxidation rate and a 50% higher reduction rate compared to WT Cyt*c* whereas both rates of oxidation and reduction of the T58I mutant were similar to those of WT and the T58A mutant (Fig. 3B,C).

Next, we tested resistance of the fully oxidized or reduced Cyt*c* variants to a high load of H_2_O_2_. Due to oxidative modifications including M80 oxidation, lysine carbonylation, tyrosine crosslinking, and others^23^, Cyt*c* can become dysfunctional and the heme group degraded, which can be monitored by measuring the spectrum of Cyt*c* showing a decrease in the absorption of the heme Soret band at 408 nm present in intact Cyt*c*. When challenged with excessive H_2_O_2_ (3 mM), T58E showed increased resistance to degradation (Fig. 3D,E). Interestingly, T58I Cyt*c* was even more resistant than phosphomimetic Cyt*c*. Both mutants also demonstrated a stronger antioxidative ability than WT and T58A Cyt*c* whereas the T58A mutant was most susceptible to rapid degradation.

### Peroxidase activity is lower in phosphomimetic cytochrome *c*

Native Cyt*c* has a low degree of peroxidase activity, which increases with the increased ratios of CL to Cyt*c*. Amplex red oxidation to resorufin was used to monitor the cardiolipin peroxidase activity of Cyt*c*. When incubated with liposomes containing a larger percentage of cardiolipin (30 and 50%), T58E Cyt*c* demonstrated a reduced cardiolipin peroxidase activity compared to WT (Fig. 3F), similar to that of T28E Cyt*c*^13^. Interestingly, T58I also showed lower peroxidase activity at the higher percentages of cardiolipin, suggesting that changes at residue 58 affect peroxidase activity of Cyt*c*.

### Mitochondrial respiration is partially inhibited in intact cells stably expressing phosphomimetic cytochrome *c*

**Figure 4:**
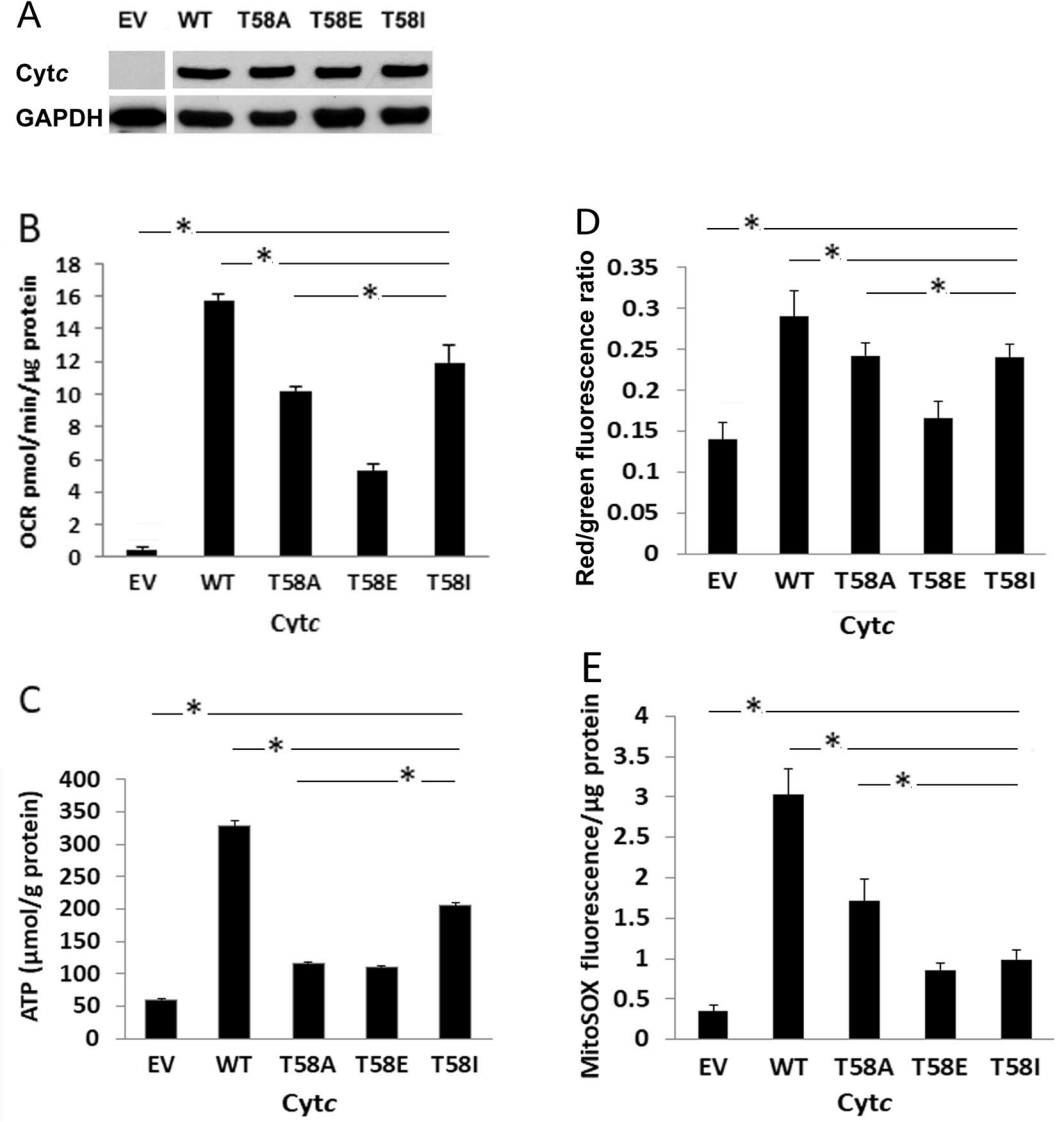
Introduction of phosphomimetic Cyt*c* into Cyt*c* knockout cells leads to a partial inhibition of respiration, intermediate mitochondrial membrane potential, and low ROS levels. (**A)** Western blot analysis was used to select the cell lines with equal expression levels of Cyt*c* variants. The blot of the empty vector was cropped from a separate gel; see the supplementary material. (**B**) OCR was measured in Seahorse medium containing 10 mM glucose using a Seahorse XF^e24^ bioanalyzer. Cells were seeded at 20,000 cells/well in 250 µL DMEM supplemented with 10% FBS and 1% penicillin and streptomycin. Data are expressed as OCR (pmol/min/µg protein). (**C**) ATP levels were assayed with the bioluminescent method. Cells (5 × 10^5^ cells/flask) were seeded in T75 flasks and harvested after overnight incubation. (**D**) Mitochondrial membrane potential was determined by JC-1 fluorescence. Cells were seeded at 15,000 per well in a Costar black 96-well plate, allowed to grow for 20 h, then incubated in phenol red-free medium containing 1 µM JC-1 for 30 min at 37 °C and analyzed via JC-1 fluorescence measurement. (**E)** Basal mitochondrial ROS was determined with MitoSOX. Cells (10^5^ cells/well) were plated in 24-well plates and incubated with MitoSOX reagent (5 µM) in phenol red-free medium. Fluorescence was analyzed using a microplate reader. Data are represented by means ± SEM. *p < 0.05.

To test the effect of phosphomimetic substitution of T58 on mitochondrial respiration in intact cells, Cyt*c* double knockout mouse embryonic fibroblasts were used to generate stable cell lines expressing Cyt*c* variants (the mouse and rat somatic Cyt*c* protein sequences are identical). Both somatic and testis Cyt*c* were knocked out to ensure that the lack of the somatic isoform does not induce the expression of the testis isoform and restore mitochondrial respiration^24^. We chose stable cell line clones with equivalent expression levels of each Cyt*c* variant (Fig. 4A) and analyzed intact cell respiration using a Seahorse bioanalyzer. If the reaction between Cyt*c* and COX is rate-limiting, reduced respiration rates seen *in vitro* should translate into reduced oxygen consumption rates in intact cells. Cell lines stably expressing T58E and T58I Cyt*c* displayed 68% and 25% reduced respiration rates, respectively (Fig. 4B), which is consistent with the results obtained with purified COX *in vitro* (Fig. 2B). T58A Cyt*c*-mediated respiration rate was between WT and T58E (Fig. 4B). In addition, ATP levels were reduced by 65%, 66%, and 36% for cells expressing T58A, T58E, and T58I, respectively (Fig. 4C).

### Mitochondrial membrane potential and ROS production are decreased in cells expressing phosphomimetic cytochrome *c*

Since a decreased mitochondrial respiration rate was observed in intact cells after phosphomimetic substitution of T58, we hypothesized that this should translate into a reduction of the mitochondrial membrane potential (ΔΨ_m_). We measured ΔΨ_m_ using JC-1, a voltage-dependent probe, and found that the ΔΨ_m_ was indeed reduced in T58E and T58I Cyt*c*-expressing cells, as indicated by ~50% and 30% decreased JC-1 fluorescence (Fig. 4D), which matches the pattern of the corresponding respiration rates. As membrane potential determines mitochondrial ROS production, we measured ROS levels in the mitochondria of our Cyt*c* variant-expressing cells using MitoSOX, a mitochondrial ROS indicator. We found that the ROS levels were also reduced, as shown by 70% and 60% decrease in fluorescence in T58E and T58I Cyt*c*-expressing cells, respectively (Fig. 4E).

### H_2_O_2_- or staurosporine-induced apoptosis is decreased in cells expressing phosphomimetic cytochrome *c*

**Figure 5:**
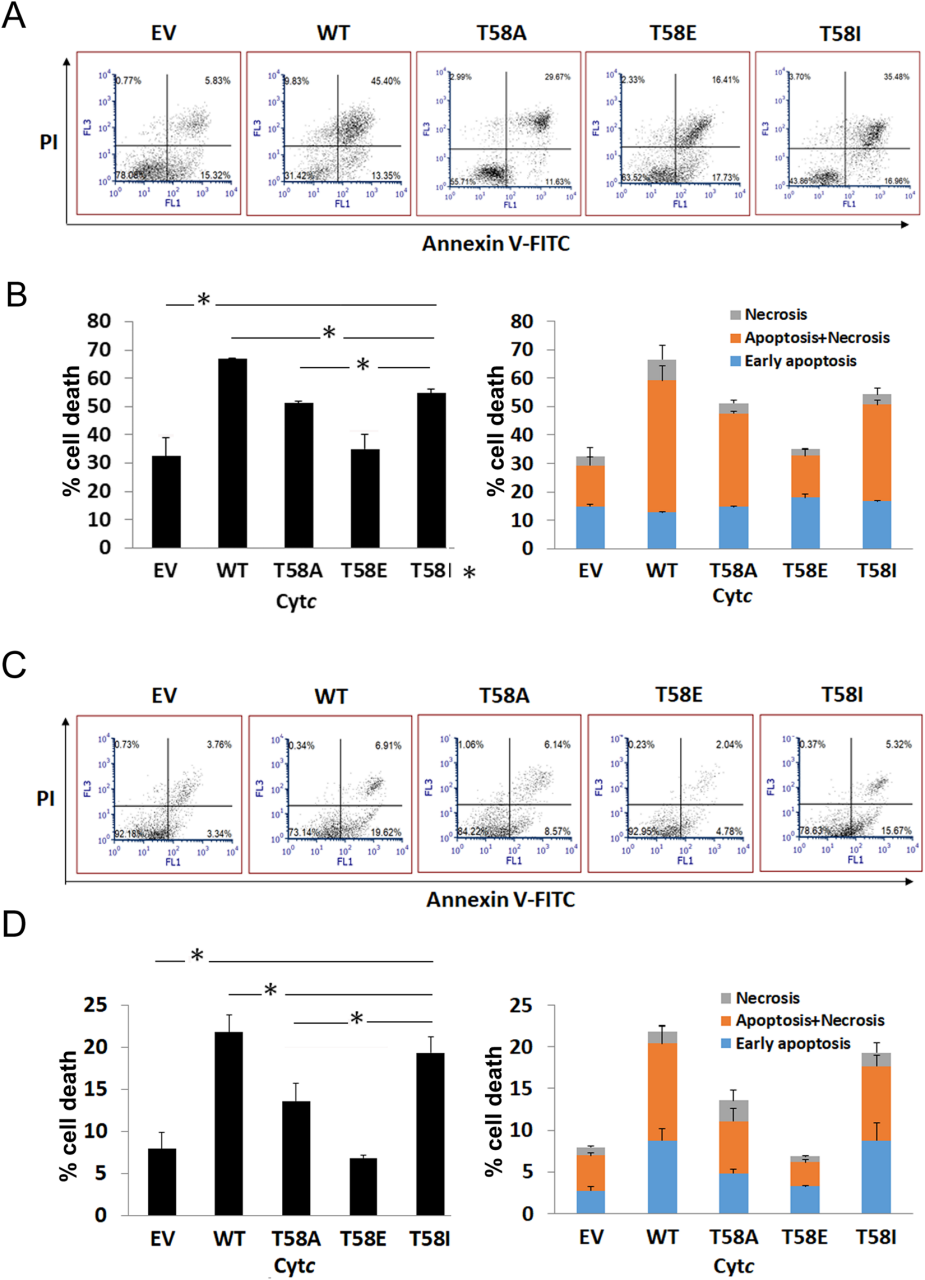
H_2_O_2_- or staurosporine-induced apoptosis was decreased in cells expressing Cyt*c* variants. Cyt*c* double knockout lung fibroblast cell lines expressing Cyt*c* variants or empty vector were treated with 300 µM H_2_O_2_ for 14 h (panels A and B), or 1 µM staurosporine for 5 h (panels C and D). The cells were incubated with both annexin V-FITC and PI, detected by FloMax flow cytometry, and analyzed with FCS Express 6 software. Viable cells were not stained by annexin and PI, which are present in the lower left quadrant, early apoptotic cells in the lower right quadrant were stained for annexin V but not PI, necrotic and/or apoptotic cells in the upper right quadrant were stained by both annexin V and PI. Panels A and C show dot plots acquired with the FloMax software. Panels B and D are quantitation of cell death (%) by apoptosis/necrosis. Data are expressed as means ± SEM. *p < 0.05.

Our *in vitro* data showed that phosphomimetic T58E Cyt*c* was able to trigger activation of caspase-3 but to a much lower extent compared to WT Cyt*c* in the cell-free detection system. To confirm that this “controlled” caspase-3 activation by phosphomimetic T58 Cyt*c* can occur in intact cells, we analyzed apoptosis induced with either H_2_O_2_ or staurosporine for all Cyt*c* mutants using the empty vector and WT cells as controls. Consistently, phosphomimetic substitution of Cyt*c* T58 resulted in a significant decrease in the rates of both H_2_O_2_- (Fig. 5A,B) and staurosporine-induced apoptosis and necrosis (Fig. 5C,D). As expected, cells transfected with empty vector displayed much lower rates of cell death compared to WT due to absence of Cyt*c*.

### Molecular dynamics simulations suggest structural and functional differences of the T58 variants

**Figure 6:**
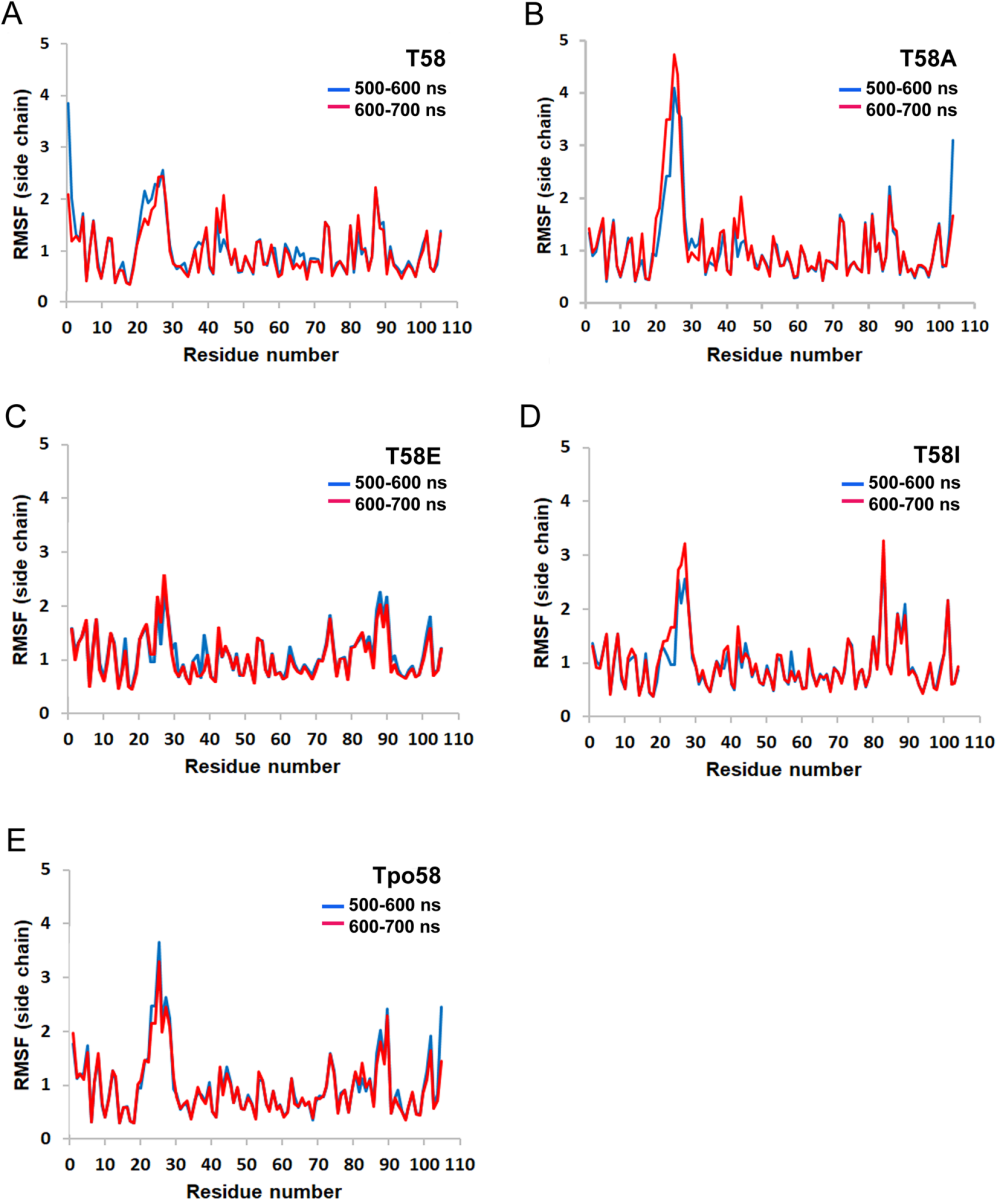
Molecular dynamics simulations. All five simulation averages were calculated for the 500–600 ns (blue) and 600–700 ns (red) intervals. The RMSF of the amino acid side chains are shown. All five runs displayed equilibrium within 500 ns to 700 ns. (**A**) Molecule A from the WT Cyt*c* crystal structure (PDB entry 5C0Z). (**B**) Equivalent to (**A**) for T58A Cyt*c*. (**C**) Equivalent to (**C**) for T58E Cyt*c*. (**D**) Equivalent to (**A**) for T58I Cyt*c*. (**E**) Equivalent to A for Tpo-T58 Cyt*c*. The RMSF values in the 20–30 loop are higher compared to other domains of the protein.

To explore possible differences of the Cyt*c* T58 variants in their solution structures, molecular dynamics simulations were performed using the A chain from WT Cyt*c*^13^ or the modeled mutated variants as well as a model of phosphorylated Cyt*c* with the phosphate group added to T58 in the WT molecule A using COOT (PDB code Tpo58). In all five simulations of the amino acid side chains, the final solution structures of Cyt*c* after 600 and 700 ns remained stable. The loop consisting of amino acids 20–30 showed the highest root mean square fluctuations (RMSF) for all variants (Fig. 6), similar to what we reported recently for Cyt*c* variants of T28^13^. The loop RMSF were the highest for T58A Cyt*c*, which may explain the structural instability of the T58A mutant when subjected to H_2_O_2_ (Fig. 3D,E).

## Discussion

Previously, we reported phosphorylation of Cyt*c* at three distinct sites with tissue specificity, Y97 in heart, Y48 in liver, and T28 in kidney. In the present study, we isolated Cyt*c* from normal rat kidney and found that threonine 58 was also phosphorylated in some Cyt*c* preparations from kidney while T28 was always phosphorylated^13^. The identification of T58 as another phosphorylation site in kidney in only some preparations could suggest that it is a minor posttranslational modification in this organ as a whole or, alternatively, that it only occurs in one or a few of the more than 26 cell types of the kidney^25^.

Since Cyt*c* isolated from tissues is usually only partially phosphorylated, and because phosphorylations are somewhat unstable and can be easily lost, especially during longer experiments, and because it is cumbersome to acquire sufficient amounts of phosphorylated Cyt*c* isolated from mammalian tissues, we here used an *in vitro* approach to test the functional effect of T58 phosphorylation. We mutated T58 to phosphomimetic glutamate, non-phosphorylatable alanine, and isoleucine to dissect the functional effects of the T58 modifications, using recombinant Cyt*c* variants and a cell culture model using Cyt*c* deficient mouse embryonic lung fibroblasts stably expressing Cyt*c* variants.

The biochemical and functional characterization of the overexpressed phosphomimetic Cyt*c*, which introduces a negative charge mimicking threonine phosphorylation, revealed that the substitution of T58 with glutamate partially inhibits mitochondrial respiration - both *in vitro* and in intact cells - and reduces apoptotic cell death. Although all Cyt*c* variants generated hyperbolic kinetics in the reactions with purified bovine liver COX, maximal turnover was decreased by 45% and 25% with the phosphomimetic and isoleucine-substituted Cyt*c* mutants compared to the WT. Similar reductions were seen in intact cells. These results are consistent with our previous findings with phospho- or phosphomimetic-Y97, Y48, and T28, except that T28 phosphorylation did not have any effect on apoptosis. Our data further support a high-throughput study which concluded that, in general, phosphorylation of metabolic mitochondrial proteins negatively regulates mitochondrial enzyme activity whereas dephosphorylation activates mitochondrial functions^26^.

The redox midpoint potentials of the four Cyt*c* variants were in a similar range, suggesting that they may only constitute a minor component to the observed functional differences, including the reaction with COX and intact cell respiration. With the lower oxidation rate and higher reduction rate, phosphomimetic T58E Cyt*c* may exhibit a slower rate of electron transfer to COX and increased ROS scavenging activity. Phosphomimetic T58E Cyt*c*, both at the fully oxidized and reduced state, displayed increased resistance to heme degradation by H_2_O_2_, possibly due to its lower oxidation rate, which may be a result of conformational changes caused by the phosphomimetic substitution.

**Figure 7:**
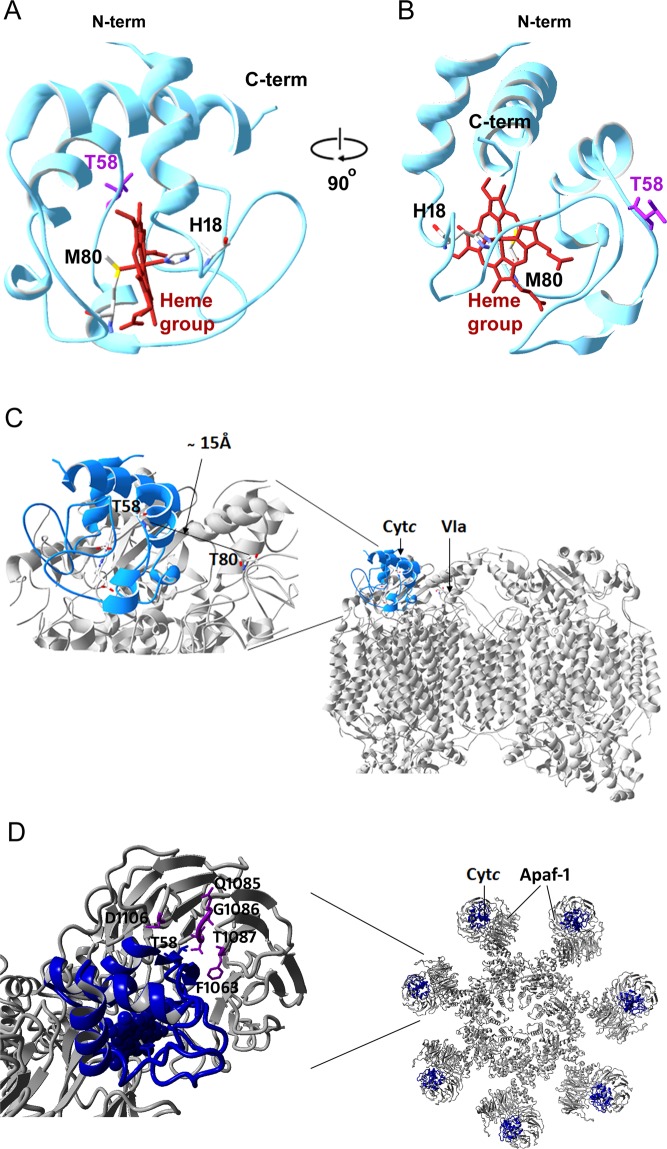
Threonine 58 location on Cyt*c* and docking models of Cyt*c* interaction with COX and Apaf-1. **(A**) Cyt*c* structure shows T58 is located on the back side of the heme crevice. Crystallographic data from rat Cyt*c* (PDB entry 5C0Z) was used^13^ and processed with the program Swiss PDB viewer (version 4.1.0). The heme group (red) is in a hexacoordinated configuration with His18 and Met80 as two axial ligands. (**A**) Conventional view, residue T58 is on the back of the molecule. (**B**) Horizontally rotated view, T58 is shown on the right side of the molecule. (**C**) Docking model of Cyt*c* interaction with COX. The Cyt*c* structure (PDB entry 5C0Z) docked onto COX was used based on the Roberts model^49^. Analysis with Swiss PDB viewer software showed that Thr58 of Cyt*c* is in the proximity of T80 of COX VIa subunit within 15 Å as the closest residue. (**D**) Docking model of a Cyt*c* -Apaf-1 binding in the apoptosome. The views are shown of the heptameric platform from the apoptosome model (PDB entry 3JBT), with top view showing of Apaf-1 subunit along with the associated Cyt*c* and Apaf-1 pair (a). A Cyt*c* and Apaf-1 pair was zoomed in and processed with Swiss PDB viewer software (version 4.1.0), Cyt*c* T58 (blue color) is in the proximity of F1063, Q1085, G1086, T1087, and D1106 residues (purple color) of the Apaf-1 molecule at a distance < 7 Å (b).

T58 is located in a loop on the back side of the heme crevice according to the Cyt*c* crystal structure (Fig. 7A,B). It is not one of the 23 positions among the 104 amino acid residues of Cyt*c*, previously proposed to be critical to the structure, function, folding, and stability of Cyt*c*^27^. T58 is not in direct contact with COX based on a Cyt*c*-COX docking model (Fig. 7C). As shown, the closest residue to Cyt*c* T58 on COX within a distance of 15 Å is T80 of COX subunit VIa. Therefore, phosphorylation of T58 likely affects the binding affinity of Cyt*c* with COX indirectly, leading to a reduction of the electron transfer rate.

Interaction with and peroxidation of cardiolipin was proposed to be a step for Cyt*c* release from the IMS into the cytoplasm during apoptosis^28^. The largely electrostatic interaction-centered A-site involves residues K72, K73, K86, and K8 whereas the L-site involves K22, K25, H26, K27, and H33, leading to partial unfolding of the protein. However, in addition to the A- and the L-site, a third interaction site (C-site), which includes residues F36, G37, T58, W59, and K60, was detected using reverse micelle encapsulation to better mimic cristae curvature^17^. Interestingly, in the latter study the interaction of Cyt*c* with CL at the three sites did not cause significant protein unfolding. T58 phosphorylation could impair the Cyt*c*-CL interaction at the C-site and the oxidation of cardiolipin, thereby hindering the release of Cyt*c*. This concept is supported by our findings showing that phosphomimetic T58E Cyt*c* has a lower CL peroxidase activity compared to WT *in vitro* and a significantly reduced apoptotic activity in intact cells. The human Cyt*c* structure (entry number: 3ZCF^23^) shows that T58 is in the proximity of K39 within a distance of 4 Å. T58 phosphorylated or phosphomimetic Cyt*c* may form a salt bridge with K39, which is one of the key sites for Apaf-1 binding^29^. The electrostatic interaction or formation of a hydrogen bond between T58E and K39 of Cyt*c* could cause conformational changes, thus suppressing apoptosome formation. Furthermore, Cyt*c* T58 is in the proximity of F1063, Q1085, G1086, T1087, and D1106 of Apaf-1 at a distance < 7 Å (Fig. 7D), which could also affect Cyt*c*-Apaf-1 binding when T58 is modified. For induction of apoptosis in cells stably expressing Cyt*c* variants, we used H_2_O_2_^30–32^ and staurosporine^33,34^. A recent study showed that the intrinsic apoptosis pathway has two subtypes, caspase-dependent and independent^35^, suggesting that Cyt*c*-knockout cells could still undergo apoptosis via a caspase-independent pathway in the absence of a trigger of the extrinsic pathway. Cells expressing empty vector, which lack Cyt*c*, showed a basal level of apoptosis after treatment with H_2_O_2_ or staurosporine, which may be explained by an alternative caspase-independent programed cell death pathway mediated by apoptosis inducing factor (AIF), mitochondrial serine protease HtrA2, and endonuclease G (endo G). Cells containing empty vector maintained a mitochondrial membrane potential due to reverse operation by adenine nucleotide translocase (ANT)^36^ and F_0_F_1_-ATPase^37^.

In all phyla across 285 Cyt*c* sequences from bacteria to mammals, threonine is the most conserved amino acid at residue 58^27^. Isoleucine is second-most abundant and is present in the mammalian Cyt*c* testis isoform and in humans, who only have a single functional Cyt*c* gene, which is ubiquitously expressed. Glutamate is present in over 10 species whereas alanine was present in one plant species. The organisms carrying glutamate at T58 are mostly plants and yeast species, that may benefit from a lower rate of apoptosis and respiration as an adaptation to their environments. The evolutionary tolerance on this residue may account for the absence of detrimental functional defects in the T58 Cyt*c* variants tested in this study. Isoleucine-substituted Cyt*c* behaves in between the WT and phosphomimetic Cyt*c* in its functions related to respiration and apoptosis. A previous study showed that somatic and testis Cyt*c* have distinct functions. For example, testis Cyt*c* had a threefold increased ability to reduce H_2_O_2_ and a fourfold increased ability to trigger apoptosis^38^. However, since rodent somatic and testis Cyt*c* differ in 15 amino acids out of the 104 present in the mature protein, a functional comparison of our T58I mutant and testis Cyt*c* cannot be made.

To explore possible protein dynamics changes in our mutants, we conducted molecular dynamics simulations. Interestingly, the T58 epitope is among the domains of Cyt*c* with the most stability and none of the T58 substitutions showed any obvious change in rigidity. However, T58A Cyt*c* produced structures in which the amino acid side chains within the 20-30 amino acid loop move more compared to the other Cyt*c* variants (Fig. 6). These findings may explain that in T58A Cyt*c* the heme group appears more accessible to react with oxidants such as H_2_O_2_ rendering the protein instable. Alanine, a small amino acid, is only present in a few species, possibly because it introduces additional flexibility at the heme crevice, and the reduction of protein stability may interfere with its multiple functions, similarly to what we reported for T28 Cyt*c*^13^. With the replacement of glutamate or isoleucine, as used here, Cyt*c* produces functional effects more similar to phosphorylated Cyt*c*. Both oxidized and reduced Cyt*c* species are rigid as shown by nitrogen-15 relaxation NMR^39^. However, certain amino acid replacements may cause local or global changes in protein dynamics. For example, this was suggested for G41S and Y48H Cyt*c*, mutations that cause thrombocytopenia in humans. Here molecular dynamics simulations suggested partial unfolding of the protein^40^, and increased dynamics within the 40–57 Ω-loop were identified by NMR, which could explain increased peroxidase activity as seen in the mutants^40^. Interestingly, our MD simulations show reduced RMSF in the 40–57 Ω-loop for T58E Cyt*c* (and modeled T58-phosphorylated Cyt*c*), suggesting increased stability of this important structural domain.

In conclusion, all four functionally characterized phosphorylated or phosphomimetic substituted Cyt*c* molecules including Y97, Y48, T28, and T58 showed partial inhibition of respiration. The results presented here further support our model that under basal physiological conditions, phosphorylation of Cyt*c* T58 maintains an optimal intermediate ETC electron flux and thus an intermediate membrane potential range that limits ROS generation^41^. Under stress, such as ischemia, Cyt*c* becomes dephosphorylated resulting in maximal ETC flux, hyperpolarization of membrane potential, and exponential increase of ROS production, leading to apoptotic cell death^10^. Our findings using intact cells suggest that a small modification of a Cyt*c* residue can control the overall flux of the ETC. Future work is needed to identify the signaling pathways, including kinases and phosphatases, that regulate Cyt*c* in physiological and pathological conditions.

## Materials and Methods

### Isolation of cytochrome *c* from rat kidney tissue

All reagents and chemicals were purchased from Sigma (St. Louis, MO) unless stated otherwise. Procedures for acquiring animal tissues were approved by the Wayne State University Institutional Animal Care and Use Committee (IACUC), and all experiments were performed in accordance with relevant guidelines and regulations. Rats were sacrificed, kidneys were removed, and Cyt*c* was extracted by the acid extraction method^12^. Briefly, kidney tissues were homogenized in 100 mM phosphate buffer, pH 4.5, and incubated at 4 °C overnight. When most cellular proteins are denatured and precipitated, Cyt*c* is extracted as a soluble protein in solution. Homogenates were centrifuged at 15,810 × g for 35 min, the supernatants decanted through cheesecloth, and the pH adjusted to 7.4 using KOH. PMSF protease inhibitor (1 mM) and phosphatase inhibitors (10 mM KF, 1 mM activated sodium orthovanadate) were added to preserve the phosphorylation state of Cyt*c*. The supernatant was centrifuged after pH adjustment for 20 min at 4 °C. A DE52 anion exchange column was equilibrated with 20 mM phosphate buffer, pH 7.4, to reach a conductivity of 3.6 mS/cm. The pH and the conductivity of the supernatant were adjusted to meet the DE52 column condition used for anion-exchange chromatography. Cyt*c* passed through the anion-exchange column since it is highly positively charged and was collected in the flow-through. The flow-through was adjusted to pH 6.5, and then applied to a CM52 cation exchange column, which was equilibrated with 30 mM phosphate buffer, pH 6.5, and a conductivity 5.5 mS/cm. Cyt*c* was bound to the CM52 column and oxidized on the column with 2 mM K_3_Fe(CN)_6_, then eluted via a step-gradient of 30, 50, 80, 120, and 150 mM phosphate buffers, pH 6.5. Finally, size exclusion chromatography was used to further purify the protein with a column equilibrated with 150 mM phosphate buffer, pH 6.5. The protein was concentrated in a vacuum centrifuge, desalted with an Amicon Ultra-15 3 kDa centrifugal filter unit (Millipore, Billerca, MA), and stored at −80 °C.

### Mass spectrometry to detect site-specific phosphorylations on purified cytochrome *c*

Phosphorylation site mapping on purified kidney Cyt*c* was performed as described^12^. Immunoprecipitated protein was subjected to tryptic digestion and enrichment of phosphopeptides with titanium dioxide (TiO_2_), then desalted via Sep-Pak C18 reversed phase chromatography and dried as described. Peptides were injected into the mass spectrometer (LTQ Orbitrap-Velos, Thermo Scientific, Waltham, MA) after electrospray ionization. MS/MS spectra were assigned to peptide sequences from the UniProt protein database and searched with the MASCOT algorithm for posttranslational modifications. Phosphopeptide spectra were manually verified.

### Mutagenesis, expression, and purification of cytochrome *c*

Rodent somatic Cyt*c* cDNA was cloned into the pLW01 expression vector^16,42^, which also contains the cDNA encoding heme lyase (CYC3), an enzyme that is not present in bacteria but needed for the covalent attachment of the heme group to apo-cytochrome *c*. The codon corresponding to T58 of the somatic rodent Cyt*c* cDNA was mutagenized to a glutamate residue (T58E) as a phosphomimetic replacement or an alanine residue as an additional unphosphorylatable control (T58A), as well as isoleucine (T58I), which is present in the rodent testis-specific isoform of Cyt*c* and human Cyt*c* at this position. These mutants were generated using the QuickChange lightning site-directed mutagenesis kit (Agilent, Santa Clara, CA) according to the manufacturer’s protocol. Briefly, the PLW01 Cyt*c* plasmid was amplified using the following mutagenesis primers. Forward primers 5′-CCAACAAGAACAAAGGTATCGAGTGGGGAGAGGATACCC-3′ (T_m_ = 69 °C), 5′-ACAAGAACAA AGGTATCGCTTGGGGAGAGGATACC-3′ (T_m_ = 67 °C) and 5′-CCAACAAGAACAAAGGTATCA TCTGGGGAGAGGATAG-3′ (T_m_ = 78 °C) and the corresponding reverse-complemented primers 5′-GGGTATCCTCTCCCCACTCGATACCTTTGTTCTTGTTGG-3′ (T_m_ = 69 °C), 5′-GGTATCC TCTCCCCAAGCGATACCTTTGTTCTTGT-3′ (T_m_ = 67 °C) and 5′-TATCCTCTCCCCAGATGAT ACCTTTGTTCTTGTTGG-3′ (T_m_ = 78 °C) were used for PCR to generate T58E, T58A, and T58I mutants, respectively. Parental DNA was digested using the restriction enzyme DpnI and mutated DNA was transformed into XL10-Gold Ultracompetent cells (Stratagene, Technologies, La Jolla, CA). Plasmids containing PLW01-Cyt*c* mutants were purified from individual colonies by using the Wizard Plus SV miniprep purification system (Promega, Madison, WI) and mutated DNA was sequenced to confirm the presence of the desired mutation. Constructs were transformed into competent *E. coli* C41 (DE3) cells (Lucigen, Middleton, WI) for protein expression^16^. The sequence-confirmed clones were cultured in 10 mL of TB medium (Difco, BD, Franklin Lakes, NJ) supplemented with 100 mg/mL carbenicillin and allowed to grow at 37 °C overnight while shaking. These cultures were inoculated in 4 L of TB medium with 100 mg/mL carbenicillin and allowed to grow until A_600_ reached 2–3. The expression of Cyt*c* was induced by addition of 100 µM isopropyl β-D-1-thiogalactopyranoside (IPTG) and the protein was overexpressed in the culture at 37 °C for 6-8 h. Cells were harvested by centrifugation at 8,400 × g, at 4 °C for 40 min, and the pellets were frozen and stored at -80 °C until use. For extraction of Cyt*c*, the bacterial pellets were resuspended in lysis buffer consisting of 20 mM phosphate, pH 7.4, supplemented with a mixture of protease inhibitors (P8340, Sigma, St. Louis, MO) according to the manufacturer’s instructions. For every 10 g of bacterial pellet, 100 mL of lysis buffer was used to resuspend the cells, which were lysed using a French pressure cell press (AMINCO, American Instrument Co.). The lysates were centrifuged at 15,000 rpm at 4 °C for 45 min, the supernatant pH was adjusted to 7.4, and Cyt*c* mutants were purified by ion exchange chromatography as described above^16^.

To express Cyt*c* mutants in Cyt*c* double knockout mouse lung fibroblasts, the pBABE-puromycin expression plasmid (Addgene, Cambridge, MA) was used with rodent Cyt*c* cloned into BamHI and EcoRI restriction sites using the following primers: outer forward primer pBABE, 5′-ATCTTGT GGAAAGGACGCGGGATCCATGGGTGAT GTTGAAAAA-3′ (T_m_ = 68.0 °C); outer reverse primer pBABE, 5′-GGTCGACCACTGTGCTGGCGAATTCTTACTTATCGTCGTCATCC TTGTAATCTTCATTAGTAGCC-3′ (T_m_ = 68.8 °C). A similar PCR mutagenesis approach was used with the same mutagenesis primers as above to obtain the C-terminal 1 x FLAG-tagged WT, T58E, T58A, and T58I constructs. The mutants were transfected into Cyt*c* double knockout lung fibroblast cells^24^ and cultured at 37 °C in DMEM (high glucose, Gibco BRL) with 10% FBS, 1% of penicillin/streptomycin, 50 mg/mL uridine, 1 mM pyruvate, and 5% CO_2_. Stable cell lines expressing WT, the three mutants, and an empty vector control were selected with 2 µg/mL puromycin.

### Concentration determination of cytochrome *c*

Purified Cyt*c* was first fully reduced with 100 mM sodium dithionite, desalted with a NAP-5 column (GE Healthcare), and absorbance was measured on a Jasco V-570 double beam spectrophotometer (2 nm bandwidth). The concentration was calculated via differential spectra at 550 nm by subtracting the oxidized form from the reduced form using the formula (A_550_-reduced - Ab_550_-oxidized) /19.6 mM × dilution factor. Cyt*c* purity was confirmed by Coomassie blue staining on a 10%Tris-Tricine SDS-PAGE gel.

### Western blot analysis

Twenty micrograms of protein from cell lysates containing overexpressed Cyt*c* were subjected to SDS-polyacrylamide gel electrophoresis (SDS-PAGE) using a 10% Tris-Tricine gel. Proteins were transferred onto a PVDF membrane (0.2 µm, Bio-Rad, Hercules, CA) in 25 mM Tris base, 192 mM glycine, and 20% methanol for 100 min at 175 mA. Western analysis was performed with a 1:5000 dilution of mouse anti-Cyt*c* (556433, 7H8.2C12, BD Biosciences, San Jose, CA), followed by 1:10,000 dilution of anti-mouse IgG conjugated with horseradish peroxidase (NA93IV, GE Healthcare, Chicargo, IL). Signal was generated using HyGLO ECL reagent (E-2500, Denville Scientific Inc., Metuchen, NJ).

### Measurement of cytochrome *c* oxidase activity

Regulatory competent bovine liver COX, the same isozyme present in kidney, was purified as described^21^ and diluted to 3 µM in COX measuring buffer (10 mM K-HEPES, pH7.4, 40 mM KCl, 1% Tween 20), containing a 40-fold molar excess of cardiolipin and 0.2 mM ATP, and dialyzed overnight at 4 °C to remove cholate bound to COX during enzyme purification. COX activity at a concentration of 150 nM was measured in a chamber equipped with a micro-Clark-type oxygen electrode (Oxygraph system, Hansatech, Pentney, UK) at 25 °C in 200 µL of COX measuring buffer and 20 mM ascorbate as electron donor. Increasing amounts of purified Cyt*c* variants (0–20 µM) were added, and oxygen consumption was analyzed using Hansatech Oxygraph software. COX activity was expressed as turnover number (sec^−1^).

### Caspase-3 activity with T58 cytochrome *c* variants

Caspase-3 activation was assayed using an *in vitro* cell-free apoptosis detection system with cytosolic extracts from Cyt*c*^−/−^ mouse embryonic fibroblasts (ATCC® CRL-2613™, Manassas, VA)^24^ as described^16^. Briefly, cells were cultured in 8 × 75 cm^2^ flasks, trypsinized, pelleted, and washed twice with cold PBS, followed by one wash with cytosol extraction buffer (CEB: 20 mM HEPES, pH 7.5, 1.5 mM MgCl_2_, 10 mM KCl, 1 mM EGTA, 1 mM EDTA, 1 mM DTT, 100 μM PMSF). One mL of CEB was added to the cell pellet and the suspension was transferred to a 2 mL Dounce homogenizer, allowing it to swell in the hypotonic CEB for 15 min on ice. Cells were disrupted by 30-35 strokes with a tight pestle. Lysates were centrifuged at 15,000 × g for 15 min at 4 °C to remove organelles and nuclei. Protein concentration was measured using the DC protein assay kit (Bio-Rad, Hercules, CA). Caspase-3 activity induced by addition of purified Cyt*c* variants was measured using the EnzChek Caspase-3 assay kit (Invitrogen, Carlsbad, CA). Rhodamine 110-linked DEVD tetrapeptide was used as an artificial substrate of caspase-3, which fluoresces upon cleavage by caspase-3. Cell extracts were used at a protein concentration of 1 mg/mL and incubated with wild-type and T58 Cyt*c* variants at 20 μg/mL for 2 h at 37 °C. Ten µL of preincubated (active) extracts were utilized for caspase-3 activity assays carried out in quadruplicate, and with caspase-3 inhibitor as control according to the manufacturer’s instructions. Fluorescence was detected using a Fluoroskan Ascent FL plate reader (Labsystems, Thermo Scientific, Waltham, MA, excitation filter 485 nm with 14 nm bandwidth, emission filter 527 nm with 10 nm bandwidth). Fluorescence values were measured in 30 min intervals for 3 h at room temperature. Fluorescence units from the caspase inhibitor and background (cytosolic extract without Cyt*c*) were subtracted. Data were expressed as a percentage of change compared to the WT.

### Thermal shift assay

Thermal shift assay was performed using an Applied Biosystems 7500 Real-time PCR system to determine thermal stability of the proteins. A buffer mimicking physiological salt concentrations (140 mM KCl, 10 mM KPi, pH 7.4) was used along with a final SYPRO orange (5000X stock, Invitrogen) concentration of 2.5X and a Cyt*c* concentration of 0.2 mg/mL. The total reaction volume was 25 µL and the microplate was sealed with an optical clear adhesive film. The thermal stability was measured as a standard curve from 25 to 95 °C with a ramp speed of 0.5 °C/min. The melting temperature of each mutant was determined using the negative first derivative curve^22^ generated by ABI 7500 Software v2.0.6.

### Measurement of redox potential

The midpoint redox potential (E°) was measured as described^13^ using 2,6-dichloroindophenol (DCIP, E°′ = 237 mV) as a reference compound, which has an absorption band at 600 nm in its oxidized state. Briefly, 1 mL of 2 mg/mL Cyt*c* solution was added into a cuvette with 50 µL of 1 mM potassium ferricyanide, 2 mL of 50 mM citrate buffer, pH = 6.5, and 0.1 mL of 1 mM DCIP. Absorbance corresponding to fully oxidized Cyt*c* (A_550_-A_570_) and DCIP (A_600_) were recorded using a Jasco V-570 double beam spectrophotometer. Then sequential 1 µL additions of 5 mM ascorbate were used to reduce Cyt*c*, and absorbance values were measured for each step in one-minute intervals. When the readings were constant, a few grains of sodium dithionite (Na_2_S_2_O_4_) were added at the end of the titration to fully reduce Cyt*c* and DCIP. The ratios of oxidized and reduced forms of Cyt*c* and DCIP were plotted as log (DCIP_ox_/DCIP_red_) versus log (Cyt*c*_ox_/Cyt*c*_red_), which yielded a linear line with a slope, *n*_DCIP_/*n*_Cyt*c*_, and a y-intercept, *n*_Cytc_/59.2 (E_Cyt*c*_-E_DCIP_). These values were used to calculate the E°_Cyt*c*_ with the Nernst equation.

### Measurements of cytochrome *c* oxidation and reduction rates

The kinetics of oxidation of ferro-Cyt*c* by H_2_O_2_ and reduction of ferri-Cyt*c* by ascorbate were measured spectrophotometrically at 550 nm as described^38^. Briefly, WT and T58E, T58A, and T58I Cyt*c* mutants were fully reduced with sodium dithionite, and the proteins were desalted using NAP5 columns. The initial spectra of 15 µM Cyt*c* in 0.2 M Tris-Cl, pH 7.0 were measured at both 550 nm and 630 nm (as background reading). Then oxidizing agent H_2_O_2_ (100 µM) was added to the cuvette and the absorbance at 550 nm was recorded at 10, 20, and 30 sec. The amount of oxidized Cyt*c* was calculated as described above. For reduction rate analysis, the kinetics of reduction of Cyt*c* with ascorbate were measured. Cyt*c* variants were fully oxidized with K_3_Fe(CN)_6_ and desalted using NAP5 columns. Ferri-Cyt*c* (15 µM) was added to 50 mM sodium phosphate, pH 7.0, and 200 µM ascorbate was added to the cuvette and sealed from air. The measurements were performed as described above.

### Heme degradation assay

The degradation of heme was analyzed through dissipation of the Soret band at 408 nm using 5 µM ferri- or ferro-Cyt*c* in 50 mM phosphate buffer, pH 6.1, with 3 mM H_2_O_2_ as described^38^. Spectra were recorded initially, after 60 sec, followed by measurements every 200 sec until 800 sec.

### Measurement of cardiolipin peroxidase activity

The cardiolipin peroxidase activity of Cyt*c* was measured as previously described^13,16^, with modifications. Fluorescence of resorufin, the oxidation product of Amplex Red, was detected using a Flouroskan Ascent microplate reader (Labsystems, Thermoscientific) with excitation and emission wavelengths of 530 nm and 590 nm, respectively. Liposomes containing 0%, 20%, 30%, and 50% of tetraoleoyl-cardiolipin (TOCL) and 1,2-Dioleoyl-sn-glycero-3-phosphocholine (DOPC) in 20 mM K-HEPES buffer, pH = 7.2 were sonicated 5 times for 30 sec on ice, with one-minute intervals. Liposomes (25 µM) were incubated in a 96-well plate with 1 µM Cyt*c* in the presence of 20 mM K-Hepes. The reaction was started with the addition of 10 µM Amplex Red and 5 µM H_2_O_2_. The reaction progress was monitored for 5 min, during which the reaction rate was linear.

### Measurement of oxygen consumption rate in intact cells

Cells were cultured in DMEM supplemented with 10% FBS, 1% penicillin/streptomycin in Seahorse XF^e24^ cell culture microplates at a density of 20,000 cells per well in 250 μL media and incubated at 37 °C and 5% CO_2_. After 20 h the medium was replaced with Seahorse XF medium supplemented with 10 mM glucose, pH 7.4, and intact cell respiration was measured with an XF^e24^ Analyzer (Seahorse Biosciences, North Billerica, MA). Normalization to total protein content in the well after the experiment was used to control for variation in cell number between the cells expressing WT and T58 Cyt*c* variants. Following the Seahorse assay, the cells in each well were lysed with 25 μL RIPA buffer containing protease inhibitor cocktail (Roche Applied Science, Mannheim, Germany) and protein concentration was determined using the DC protein assay (Bio-Rad, Hercules, CA), standardized to a bovine serum albumin (BSA) standard curve.

### Measurement of mitochondrial membrane potential

Cell lines stably expressing Cyt*c* variants were seeded at a density of 20,000 cells/well onto black 96-well plates (Costar, CLS3603, Sigma, Ronkonkoma, NY) and allowed to grow to 80–90% confluence. To assess relative changes in ΔΨ_m_, cells were incubated for 1 h in phenol red- and FBS-free medium containing 1 µM JC-1 (5,5′,6,6′-tetrachloro-1,1′,3,3′-tetraethyl-benzimidazolyl-carbocyanine iodide, Molecular Probes, Inc.). JC-1 can selectively enter mitochondria and exists as a monomer at low membrane potential or at low concentration, emitting green fluorescence. At higher membrane potential, JC-1 aggregates and emits red fluorescence. Fluorescence was measured in PBS using a Synergy H1 microplate reader (BioTek Instruments, Inc., Winooski, VT) with excitation: 485 nm; emission: 527 nm, and excitation: 485 nm; emission: 590 nm, respectively. Data were presented as the ratio of red to green fluorescence.

### Mitochondrial ROS measurement

Cells expressing Cyt*c* variants were cultured in 24-well plates and incubated with 5 µM MitoSOX (M36008, Thermo Scientific) for 30 min at 37 °C. Cells were washed with PBS, and fluorescence was analyzed with a Synergy H1 plate reader (BioTek, Winooski, VT) by using excitation and emission wavelengths of 510 nm and 580 nm.

### ATP Assay

Lung fibroblast cells stably expressing Cyt*c* variants cultured in a T75 flask were scraped in warm 1 × PBS, collected as triplicates and immediately stored at −80 °C until measurement. Release of ATP was performed by boiling the samples in 300 µL boiling buffer (100 mM Tris-Cl, pH7.75, 4 mM EDTA) and immediate transfer of the samples to a boiling water bath for 2 min. Samples were kept on ice, sonicated, and diluted by 300-fold, and 40 µl of the diluted samples were used to determine the ATP concentration with the ATP bioluminescence assay kit HS II (Roche Applied Science) following the manufacturer’s protocol. Data were normalized to the protein concentration.

### Apoptosis of stable cell lines expressing cytochrome *c* variants

Apoptosis was detected by staining the cells with Annexin V-FITC and propidium Iodide (PI) dyes, following H_2_O_2_ treatment 300 μM^43–45^ for 14 h or 1 µM staurosporine treatment^46^ for 5 h, respectively, with modifications. Briefly, cells were detached using 1 mM EDTA in PBS at 37 °C for 1 min. The reaction was neutralized by adding 2 mL of medium to the cells. Cells were pelleted and washed twice with cold PBS and resuspended in 1x Binding Buffer (0.1 M HEPES, pH 7.4, 1.4 M NaCl, and 25 mM CaCl_2_ solution) at a concentration of ~1 × 10^6^ cells/mL. The cell suspensions (100 µL; ~10^5^ cells) were transferred to a 5 mL culture tube, 5 µL of Annexin V and 2 µL PI were added to the cells as described in the Annexin V apoptosis kit manual (BD Biosciences). The cells were gently mixed and incubated for 15 min in the dark at room temperature. Binding buffer (500 µL) was added to each tube and the data were acquired by flow cytometry (FloMax, Sysmex America, Inc., Lincolnshire, IL) within 1 h. Unstained and stained/untreated cells were analyzed as controls for H_2_O_2_-treated samples whereas the same amount of DMSO vehicle was used as control for staurosporine-treated samples. The data were analyzed using FCS Express 6 RUC software (De Novo Software, Glendale, CA).

### Molecular dynamics

Molecular dynamics were performed with YASARA version 17–12–24^47^ using its conservative “slow” protocol and the recommended default forcefield, AMBER 2014^48^. The starting structure for the molecular dynamics calculations on WT is from molecule A in 5C0Z.PDB, which was obtained from oxidized Cyt*c* in the presence of potassium ferricyanide (K_3_[Fe(CN)_6_]), and T58 mutants were all modeled from WT molecule A. RMSF plots for the amino acid side chains were generated with Excel using data imported from YASARA.

### Statistical analyses

Statistical analyses of the data were performed with MSTAT version 5.4 (University of Wisconsin, N. Drinkwater) using the Wilcoxon rank sum test. Data are reported as means ± SEM. and were considered statistically significant (*) with p < 0.05.

## Supplementary information

Supplementary Information
