# Supplementary material for: Regulation of Respiration and Apoptosis by Cytochrome c Threonine 58 Phosphorylation
Source: Sci Rep. 2019 Nov 1;9:15815. doi: 10.1038/s41598-019-52101-z (PMC6825195; doi:10.1038/s41598-019-52101-z)
Supplement: Supplementary file 1 — Supplementary Information [file 41598_2019_52101_MOESM1_ESM.docx]

**Supplementary Information for Regulation of Respiration and Apoptosis by Cytochrome *c* Threonine 58 Phosphorylation**

Junmei Wan^1,2^, Hasini A. Kalpage^1^, Asmita Vaishnav^1,2^, Jenney Liu^1^, Icksoo Lee^1,3^, Gargi Mahapatra^1,7^, Alice A. Turner^1,2^, Matthew P. Zurek^1^, Qinqin Ji^4^, Carlos T. Moraes^5^, Maurice-Andre Recanati^1,6^ Lawrence I. Grossman^1^, Arthur R. Salomon^4^, Brian F.P. Edwards^2^, Maik Hüttemann^1,2,*^

^1^Center for Molecular Medicine and Genetics, Wayne State University, Detroit, MI 48201, USA.

^2^Department of Biochemistry, Microbiology and Immunology, Wayne State University, Detroit, MI 48201, USA.

^3^College of Medicine, Dankook University, Cheonan-si, Chungcheongnam-do 31116, Republic of Korea.

^4^MCB Department, Brown University, Providence, RI 02912, USA.

^5^Department of Neurology, University of Miami School of Medicine, Miami, FL 33136, USA.

^6^Department of Obstetrics and Gynecology, Wayne State University, Detroit, MI 48201, USA.

^7^Current address: Department of Internal Medicine, Section on Gerontology and Geriatric Medicine, Wake Forest University Health Sciences, Winston-Salem, NC 27157, USA.

*Correspondence and requests for materials should be addressed to M.H. (email: mhuttema@med.wayne.edu)

|  | WT | T58A | T58E | T58I |
| --- | --- | --- | --- | --- |
| Melting Temperature (ºC) | 55.7±0.3 (T_m1_) | 54.4±1.1 (T_m1_) | 56.4±0.9 (T_m1_) | 91.5±0.2 (T_m1_) |
|  | 91.2±0.1 (T_m2_) | 91.5±0.4 (T_m2_) | 91.4±0.5 (T_m2_) |  |

**Supplementary Figure S1**

The melting temperatures (T_m_) of WT, T58A, T58E, and T58I Cyt*c* based on thermal shift assay


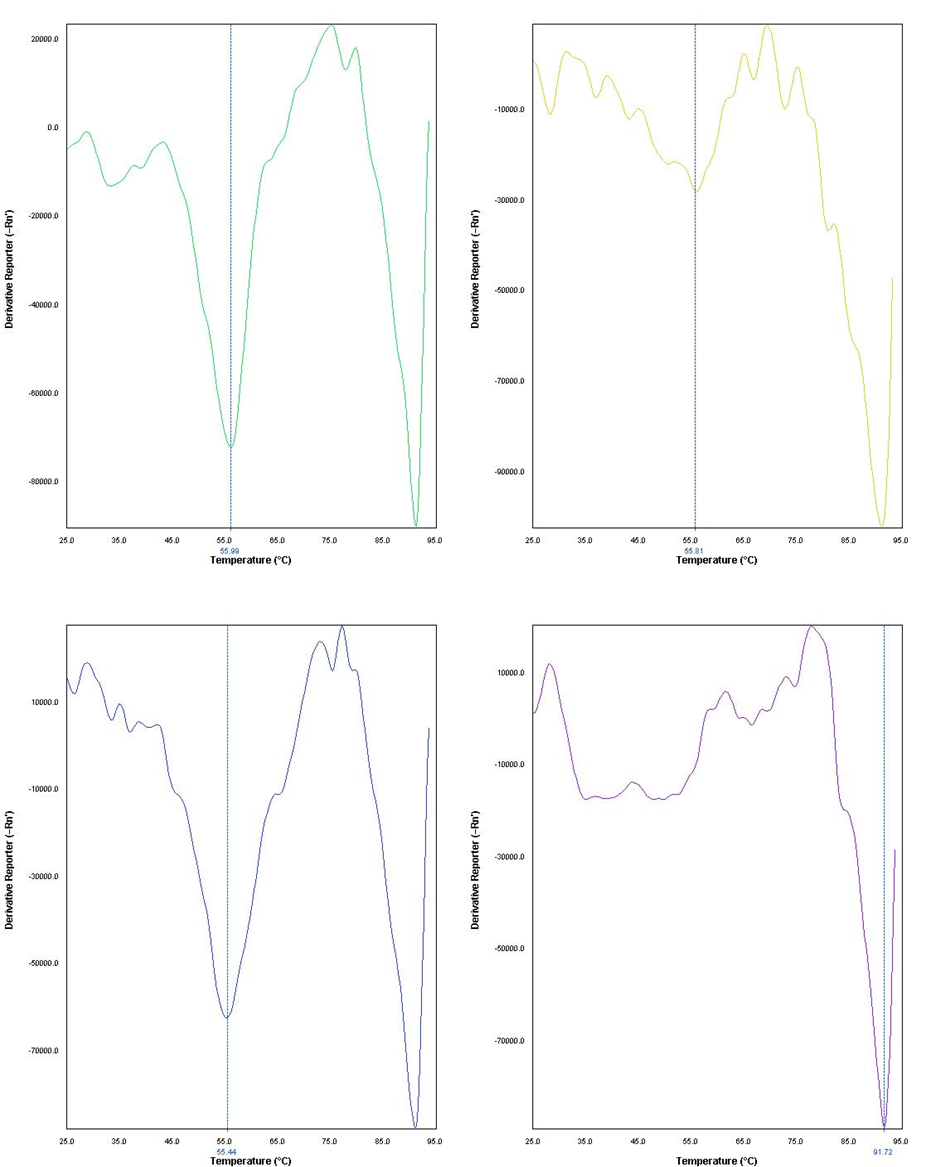


WT

T58A

T58E

T58I

**Supplementary Figure S1.** The negative first derivative melting curves of WT (green), T58A (yellow), T58E (blue) and T58I (purple). The first melting temperature (T_m1_) of each mutant is shown in blue. Supplementary table S1 summarizes the melting temperatures as mean ± s.d. from 3-5 replicates.

**Supplementary Figure S2**

X-Ray films for Western blot analysis of Cyt*c* expression shown in Figure 4A. Relevant lanes are within the boxed area enclosed by red dots including empty vector (EV) as a control. EV cell lysate was probed with, A, mouse anti-Cyt*c* and, B, mouse anti-GAPDH. Cell lysates of Cyt*c* variants were probed with, C, mouse anti-Cyt*c* and, D, mouse anti-GAPDH.

**
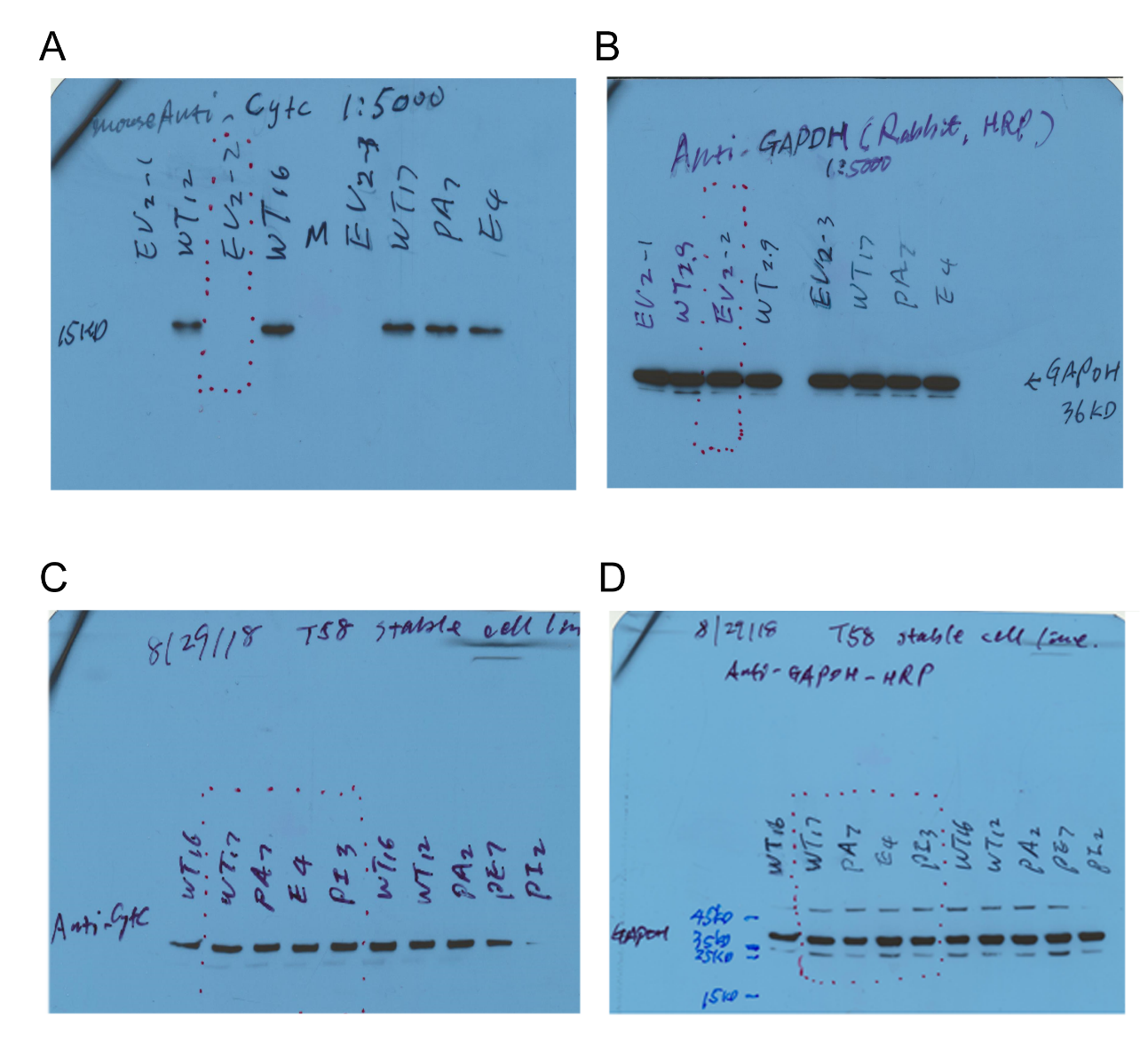
**

Fig. S2A. Full length blots for Cyt*c* in the stable cell line lysate of the EV. The band was cropped from the area within dotted line frame in red. The labels are our internal individual clones: EV2-2 refers to one of the EV cell lines. S2B. Full length blots for GAPDH detected in the stable cell line lysate of the EV. The labels are the same as S2A. S2C. Full length blots for Cyt*c* detected in the lysate of cells transfected with Cyt*c* variants. The bands were cropped from the area within the dotted line frame in red. The labels are our internal individual clones: WT17 refers to WT Cyt*c* clone 17. PA7: T58A clone 7; E4: T58E clone 4; PI3: T58I clone 3. S2D. Full length blots for Cyt*c* variants in the stable cell line lysate. The bands were cropped from the area within the dotted line frame in red. The labels are the same as in S2C.
